# A review of diamond dosimeters in advanced radiotherapy techniques

**DOI:** 10.1002/mp.17370

**Published:** 2024-09-02

**Authors:** Christina Angelou, Ileana Silvestre Patallo, Daniel Doherty, Francesco Romano, Giuseppe Schettino

**Affiliations:** ^1^ Department of Physics University of Surrey Guildford UK; ^2^ Radiotherapy and Radiation Dosimetry National Physical Laboratory (NPL) Teddington UK; ^3^ Istituto Nazionale di Fisica Nucleare (INFN) Sezione di Catania Catania Italy

**Keywords:** advanced radiotherapy techniques, diamond detector, dosimetry

## Abstract

This review article synthesizes key findings from studies on the use of diamond dosimeters in advanced radiotherapy techniques, showcasing their applications, challenges, and contributions to enhancing dosimetric accuracy. The article explores various dosimeters, highlighting synthetic diamond dosimeters as potential candidates especially due to their high spatial resolution and negligible ion recombination effect. The clinically validated commercial dosimeter, PTW microDiamond (mD), faces limitations in small fields, proton and hadron therapy and ultra‐high dose per pulse (UHDPP) conditions. Variability in reported values for field sizes <2 × 2 ${\mathrm{cm}}^{2}$ is noted, reflecting the competition between volume averaging and density perturbation effects. PTW's introduction of flashDiamond (fD) holds promise for dosimetric measurements in UHDPP conditions and is reliable for commissioning ultra‐high dose rate (UHDR) electron beam systems, pending the clinical validation of the device. Other advancements in diamond detectors, such as in 3D configurations and real‐time dose per pulse x‐ray detectors, are considered valuable in overcoming challenges posed by modern radiotherapy techniques, alongside relative dosimetry and pre‐treatment verifications. The studies discussed collectively provide a comprehensive overview of the evolving landscape of diamond dosimetry in the field of radiotherapy, and offer insights into future directions for research and development in the field.

## 1 INTRODUCTION

Accurate and traceable dosimetry is a cornerstone of modern radiotherapy as both tumor volumes and healthy tissues respond significantly to small variations in the absorbed dose. Dosimetry precision, therefore, directly impacts treatment efficacy and minimizes potential side effects for patients. Traceability, achieved through standardized calibration procedures and rigorous quality assurance measures, allows for comparisons across different treatment facilities and ensures consistency in dose delivery. It also facilitates adherence to regulatory requirements and guidelines, promoting patient safety and confidence in treatment outcomes. Without accurate and traceable dosimetry, the risk of under‐dosing or over‐dosing patients increases, potentially compromising treatment efficacy and patient well‐being. Thus, robust dosimetry practices is essential for optimizing radiotherapy outcomes and advancing patient care.

Technological advancements in radiotherapy allow for new types of megavoltage (MV) delivery techniques, such as intensity modulated radiotherapy (IMRT), volumetric arc therapy (VMAT), stereotactic body radiotherapy (SBRT), stereotactic radiosurgery (SRS), and proton beam therapy, which are more therapeutically beneficial by achieving higher dose conformity to the tumour volume and enhanced sparing effect to healthy tissues. Such techniques use non‐uniform/small radiation fields, time/space varied dose rates and high dose gradients, all of which have increased the need for accuracy and precision in the treatment process and specially on clinical dosimetry.

Special conditions for reference dosimetry in radiotherapy devices that are unable to produce agreed reference measurement conditions are presented by the IAEA TRS‐483 Code of Practice (CoP).^1^ According to Alfonso et al.,^2^ in the dosimetry of more clinically relevant fields and specifically for small MV photon fields (<3 × 3 ${\mathrm{cm}}^{2}$), the detector specific output correction factor, $k_{Q_{clin},Q_{ref}}^{f_{clin},f_{ref}}$, should be considered when calculating the absorbed dose in water. This accounts for differences between the conditions of detector size, phantom material, depth or distance, beam quality Q, field size and so on, in a clinical field $f_{clin}$ and in a reference field $f_{ref}$.^3^, ^4^ The field output factor, $\Omega_{Q_{clin},Q_{ref}}^{f_{clin},f_{ref}}$, is defined as a ratio of the absorbed dose to water in $f_{clin}$ to that in $f_{ref}$, usually that being at 10 × 10 ${\mathrm{cm}}^{2}$ field size, determined by:

(1) $$\Omega_{Q_{clin},Q_{ref}}^{f_{clin},f_{ref}}=\frac{M_{w,Q}\left(f_{clin}\right)}{M_{w,Q}\left(f_{ref}\right)}k_{Q_{clin},Q_{ref}}^{f_{clin},f_{ref}}$$

where $M_{w,Q}$ are the detector readings at the two field sizes at the reference depth, $z_{ref}$, in water. The ratio of the two readings is referred to as the output factor (OF). For broad photon beams in conventional radiotherapy, $k_{Q_{clin},Q_{ref}}^{f_{clin},f_{ref}}=1$, due to the negligible dependence of stopping‐power ratios and perturbing variables on field size.^1^ If $k_{Q_{clin},Q_{ref}}^{f_{clin},f_{ref}}$ is less than unity then the detector‐dose to water‐dose ratio is greater in the small field investigated when compared to the large field used and so an over‐response is displayed. If $k_{Q_{clin},Q_{ref}}^{f_{clin},f_{ref}}$ is greater than unity then an under‐response is exhibited.

Currently, most radiotherapy linacs are capable of delivering flattening filter free (FFF) beams. Numerous published papers emphasize their benefits, particularly their capacity to deliver greater dose rates than conventional beams with flattening filters (FF).^5^, ^6^ Higher dose rates offer the advantage of shortening treatment times. The increased interest in the implementation of radiotherapy with FFF beams means that research on the detector's response to high dose rates (up to 30 Gy/min) is a major focus, including how the field size affects their response.^7^

Furthermore, FLASH radiation is another cutting‐edge modality of radiotherapy delivery that is gaining ground and researchers' interest. The “FLASH” effect was first introduced in 2014 by Favaudon et al.^8^ The study^8^ reported that high dose rate irradiation resulted in reduced damage to normal tissue while maintaining a high tumor control probability compared to conventional radiotherapy. Numerous subsequent studies have been published that support these findings. Protons,^9^ electrons,^10^ and x‐rays^11^ have all proven to be effective at producing the “FLASH” effect, ultra‐high mean dose rate values greater than 40 Gy/s delivered in less than 500 ms. Comprehensive reviews and recent developments on the “FLASH” subject can be found in Maxim et al.^12^ and Lin et al.^13^ Dosimetry of FLASH microbeam radiotherapy (MRT) is still at an earlier stage. FLASH MRT offers a promising enhancement to conventional FLASH by introducing spatial segmentation of the dose distribution while preserving UHDRs up to 16 kGy/s. Using 50 to 500 keV x‐rays, it divides the radiation field into thin planes, a few tens to a few hundred microns wide, each being spaced apart by a few hundred microns to a few millimeters. Such spatial fractionation is believed to be responsible for considerable sparing effect and high spatial resolution dosimetry systems are required to accurately assess the unique dose distribution patterns.^14^

The conditions required for the delivery of these techniques are more complex than the ones encountered during conventional treatments and, as such, present challenges, especially to well‐established reference dosimetry protocols. Three main limitations of conventional dosimeters with regard to these modalities are the detector size, detector packaging and ion recombination effect. To address the new dosimetric requirements, both new detectors and procedures need to be considered.

Diamonds have been studied as medical dosimeters for decades due to a number of attractive properties, such as a similar atomic number to that of typical human tissues, their potential for great spatial resolution, radiation hardness, almost temperature and energy independence. In the early 2000s, developments in commercial synthetic diamonds started, which allowed diamond dosimeters to be more easily reproduced and at a lower cost.^15^, ^16^ Synthetic diamond detectors are described as reliable and robust detectors for conventional dosimetry, small field dosimetry as well as promising in UHDRs. Currently, there is only one clinically validated diamond detector, the PTW 60019 microDiamond, for which certain limitations under very small fields (<2 × 2 ${\mathrm{cm}}^{2}$) and UHDRs (>40 Gy/s) have been reported.^17^, ^18^

In this review, different types of dosimeters are introduced and compared, followed by an evaluation of the performance of commercial and non‐commercial diamond dosimeters in small photon beams, FFF photon beams, FLASH and proton and hadron beams for external beam radiotherapy (EBR). In addition, advancements in diamond detectors through other forms of application in modern radiotherapy techniques are explored.

## 2 DOSIMETER COMPARISON OVERVIEW

Stable detectors for small fields, and a wider range of dose rate and dose per pulse (DPP) than those typically considered, are essential for dosimetry in FFF beams and FLASH. Different types of dosimeters respond differently to changes in beam parameters such as dose rate, linear variation of dose, energy spectrum, field size, with diamond dosimeters showing several advantages over others. Table 1 summarizes the advantages and drawbacks of different types of commercial dosimeters in the context of small field dosimetry, and, similarly, Table 2 showcases important properties of clinically valid dosimeters with regard to high and ultra‐high DPP conditions.

**TABLE 1:** Advantages and drawbacks of commercial dosimeters for small field dosimetry.

Dosimeter type	Advantages	Drawbacks	Under‐ or over‐response	References
Radiochromic films	Near energy independence High spatial resolution Near tissue equivalence	Passive read‐out Time‐consuming lL intensive post‐processing	N/A	[1, 16, 19, 20]
Thermoluminescent dosimeters (TLDs)	Near tissue equivalence	Passive read‐out Time‐consuming Labor intensive post‐processing Energy dependence	Under‐response	[1, 21, 22]
Calorimeters	Real‐time read out Repeatability Accuracy	Low spatial resolution Many correction factors Time‐consuming	Under‐response	[23, 24, 25, 26]
Scintillators	Real‐time read out Near tissue equivalence Near energy independence High spatial resolution	Cherenkov light radiation correction	N/A	[1, 27, 28, 29, 30]
Ionization chambers	Real‐time read out Easy usage Availability Repeatability	Low spatial resolution Many correction factors	Under‐response	[31, 32, 33]
Silicon diodes	High sensitivity High spatial resolution Real‐time read out High radiation hardness	Non‐tissue equivalence Energy dependence Directional dependence (apart from edgeless diodes)	Under‐response for shielded diodes, over‐response for unshielded diodes	[16, 32, 34, 35, 36, 37, 38, 39]
Synthetic diamond dosimeters	Real‐time read out High radiation sensitivity High spatial resolution near tissue equivalence near energy independence high radiation hardness	High density sensitive volume Volume averaging below 0.7 cm field width	Under‐response and over‐response reported	[17, 37, 40, 41]

**TABLE 2:** Important characteristics of clinically valid dosimeters for high and ultra‐high DPP.

Dosimeter type	Beam type	DPP dependence	Temporal resolution	Spatial resolution	References
Radiochromic films	Electrons, protons	Independent (∼15 × 10 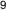 Gy/s) within 5%	Passive	A few tens of μm	[9, 27, 42, 43, 44, 45]
Thermoluminescent dosimeters (TLDs)	electrons	Independent (∼4 × 10 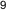 Gy/s) within 2%	Passive	A few mm	[27, 42, 43, 55]
Calorimeters	Electrons, protons	Independent (∼5 Gy/pulse, τ = 100 ns) within 1%	A few ms	A few tens of mm	[23, 24, 25, 26, 56]
Scintillators	Electrons, protons	Independent (∼4 × 10 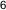 Gy/s) within 1%	∼ns	A few mm	[9, 18, 25, 27, 51, 53]
Ionization chambers	Electrons, protons	Dependent (>1 Gy/pulse, τ = 2.5 μs)	∼ms	A few mm	[9, 23, 24, 27, 42, 43, 45, 57]
Silicon diodes	Electrons	Dependent (>0.1 Gy/pulse, τ = 2.5 μs)	∼ms	A few mm	[18, 27, 54, 58]
Synthetic diamond dosimeters	Electrons, protons	Dependent(>0.1 Gy/pulse, τ = 2.5 μs)	∼ μs	A few mm (or 1 μm)	[9, 18, 27, 59]

Passive dosimeters are commonly used in radiotherapy, an example being film dosimeters. Radiochromic films are characterized as appropriate for the MV beam range due to their near energy independence and good spatial resolution (∼25 μm).^1^ For these reasons they are commonly identified as optimal detectors for small field dosimetry in MV photon beams, accurate for quality assurance (QA) of SRS and SBRT^19^ and used as reference to calculate the output correction factors for other dosimeters.^20^ Depending on the post‐processing and analysis protocols, the time needed for the self‐developing process can take between several hours and a few days. There is also the need to establish calibration curves and validate the readout system, contributing to this time‐consuming process. Another major drawback for radiochromic films is their expensive manufacturing, and therefore the cost to the user is high.^16^

Thermoluminescent dosimeters (TLDs) have advantages in absorbed dose determinations in small fields, including linear dose response up to 1 Gy and near tissue equivalence for LiF TLDs, such as LiF: Mg, Ti (TLD‐100) and LiF: Mg, Cu, P (GR‐200), but there are also remarkable limitations to their use.^1^, ^21^, ^22^ These include the cost of the reading system, time commitment, energy dependence for ${\mathrm{Al}}_{2}O_{3}$ TLDs, and lengthy waiting times before reading.^21^ TLDs, such as TLD‐100, have also demonstrated to be ineffective for measurements in field widths of <10 mm possibly due to the averaging volume effect and poor spatial resolution.^22^ Overall, even though TLDs are included in TRS‐483 CoP there are many drawbacks, and special care is required when used in small fields.

Radiochromic films and TLDs have also been studied under high DPP and ultra‐high DPP (FLASH) conditions.^42^, ^43^ Most commissioning procedures for systems in FLASH radiotherapy used in preclinical experiments are completed by passive dosimeters, such as GafChromic films, due to their dose rate independence up to ∼15 × 10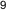 Gy/s.^27^, ^44^, ^45^ Another technique commonly employed as a reference for absorbed dose determination in UHDR electron beam systems involves the ionization of crystalline alanine.^23^, ^43^, ^46^ Alanine dosimeters generate free radicals which are detected by electron paramagnetic resonance (EPR) spectroscopy. This technique has been demonstrated to be an accurate dosimetry system for assessing the absorbed dose to water in electron beams from 0.15 to 6.2 Gy/pulse, with a pulse duration (τ) of 2.5 μs, within 0.85%.^46^ However, due to the laborious procedures previously mentioned, the design of active detectors capable of measuring dose in real‐time at extremely high DPP is crucial for quality control. Aluminum‐ and graphite‐based calorimeters have already been investigated as reference dosimeters,^23^, ^24^ but their size (20 and 7 mm in diameter and 2 and 7 mm in height, respectively) prevents them from having the high spatial resolution that is required when small fields are involved.

Another type of luminescent dosimeters are the scintillators, known for their long‐term stability, reproducibility, water equivalence and near energy independence (in case of organic scintillators), and the ability to be manufactured in small sizes (∼1 ${\mathrm{mm}}^{3}$ or less).^1^, ^18^, ^28^, ^47^ Experimental and Monte Carlo (MC) simulation studies show that the Standard Imaging Exradin W1 and W2 plastic scintillators have unity output correction factors and minimal volume averaging effects (<1%) for fields down to 0.5 × 0.5 ${\mathrm{cm}}^{2}$.^1^, ^28^, ^29^, ^30^, ^48^, ^49^ The Exradin W1 exhibits up to a 4% correction in OFs when oriented perpendicularly to the beam for such small fields.^48^ Correcting for Cerenkov radiation in the sensitive volume of the detector and optical fiber should be accounted,^28^ and there are variations in the correction factor depending on the detector's orientation within the irradiation field.^50^

Scintillators have been investigated in FLASH conditions for both electron and proton beams. Favaudon et al.^51^ used a scintillator to measure field homogeneity and width in a 5‐MeV electron beam, at dose rates from 0.4 to 3.5 × 10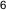 Gy/s. The detector responded linearly with a stable and reproducible signal.^51^ Similarly, another plastic scintillator demonstrated a linear response, with the accuracy of the measured absorbed dose in a 16 MeV electron beam at an average dose rate of 100 Gy/s being within ∼1%. The results agree with those of the GafChromic film within the experimental uncertainty (∼3.5%).^52^ Scintillators have also been used to measure the homogeneity of dose distribution in a 224 MeV proton beam with 100 Gy/s dose rate.^53^ In irradiation conditions with high linear energy transfer (LET) particles, referring to the average amount of energy deposited per unit track length, the scintillator detector signal is non‐linear to the absorbed dose.^25^

Standard air‐filled ionization chambers are the “backbone” of conventional radiotherapy because of their ease of use, availability and repeatability. However, they are not suitable for small field dosimetry due to the dominant volume averaging effect caused by their large detection volume, >0.3 ${\mathrm{cm}}^{3}$.^31^, ^32^ For mini‐ionization chambers, such as the PTW 31014 PinPoint chamber (active volume of 0.015 ${\mathrm{cm}}^{3}$), specifically designed for dose measurements in small fields, the volume averaging effect is still evident in fields smaller than 1.8 × 1.8 ${\mathrm{cm}}^{2}$, reaching up to 6.8% at 0.6 × 0.6 ${\mathrm{cm}}^{2}$.^33^ Investigating the $k_{Q_{clin},Q_{ref}}^{f_{clin},f_{ref}}$ values of various dosimeters using a a CyberKnife system resulted in unavoidable correction factors even for a microionization chamber, Exradin A16 (active volume of 0.007 ${\mathrm{cm}}^{3}$). For the smallest field size studied, a 0.5‐cm diameter cone, there was an underestimation of 9.9%, mainly caused by the lower density air cavity.^50^ However, as expected, there is a slightly improved performance when utilizing this microionization chamber compared to a mini‐ionization chamber, the same PinPoint ionization chamber mentioned above, as suggested by the underestimated $k_{Q_{clin},Q_{ref}}^{f_{clin},f_{ref}}$ value of 10.7% reported for the 0.5‐cm diameter cone.^50^ In addition, correcting for the ion recombination effect is not suitable when performing measurements in small fields, where the ion recombination correction and DPP have a considerable position dependence.

For ionization chambers, recombination and polarization effects are enhanced with increasing accumulated dose. DPP values above 0.1 Gy/pulse cause a decrease in ion collection efficiency and this is especially restrictive above 1 Gy/pulse (τ is 2.5 μs).^18^, ^27^, ^45^ For instance, in a study by McManus et al.,^24^ the charge collection efficiency of a parallel plate ionization chamber was found to be reduced to 4% when 5.26 Gy/pulse, with τ equal to ∼100 ns, was applied using electron beams. The spatial resolution and electrode spacing limit their suitability for dosimetric measurements in UHDPP conditions.

Considering their small sensitive volume (<0.2 ${\mathrm{mm}}^{3}$) and that only 3.6 eV are required to create a charge carrier pair in silicon versus 34 eV in air, silicon diodes have higher sensitivity than ionization chambers.^34^, ^54^ Due to their smaller sensitive volumes they also show better spatial resolution. Silicon diodes have a linear response with dose and high radiation hardness for conventional beams. However, due to silicon's high atomic number (*Z* = 14), there is a strong variation in the mass energy absorption coefficient and electron stopping power of silicon compared to that of water.^16^ They, therefore, show an energy dependence and the measured absorbed dose represents an overestimation as a consequence of the higher sensitivity to the interaction from the low‐energy component of the photon spectrum. Shielded silicon diodes have found a place in relative dosimetry, as the low energy component of the photon spectrum is attenuated by the high atomic number of shielding material used as part of the detector's encapsulation.^32^

Nevertheless, in small MV fields, there is a smaller contribution to the dose from the low‐energy scatter radiation. Consequently, the silicon response to the scattered lower energy component of the spectrum is less important and so, adding a high atomic shielding material can produce an underestimation of the dose.^38^ Thus, for small fields the use of unshielded diodes is preferred, although energy dependence correction factors should still be considered. A further limitation of silicon diodes is their directional dependence due to their construction and geometry. This needs to be taken into consideration when measuring beam profiles and percentage depth dose curves (PDDs) since the angular distribution of scattered photons and electrons changes with distance and depth from the central axis.^35^ Recently, edgeless diodes have been introduced with a negligible angular dependence within ±1.5% up to $\pm 180^{\circ }$, but the required dose rate correction factor may limit their use for patient‐specific QA.^39^ The “Edge‐on” MOSkin detectors, metal oxide semiconductor field effect transistors (MOSFET) with a spatial drop‐in packaging, represent another example of solid‐state dosimeters. The sensitivity of these detectors is independent of the lateral dimensions of their small sensitive volume (0.002 ${\mathrm{mm}}^{3}$), and their OF measurements are in good agreement with those of GafChromic film, with a 1.9% deviation, for cone diameters down to 4 mm in MV beams.^60^

Synthetic diamond dosimeters fulfil most of the characteristics of an ideal dosimeter in conventional and non‐conventional radiotherapy. Results reporting good performance and reliability of this type of dosimeter in multiple studies have increased the interest in this technology.^40^, ^61^, ^62^, ^63^, ^64^ Diamonds have a high radiation sensitivity, and hence very small diamonds, of the order of a few cubic millimeters, can be manufactured. They can be considered water equivalent as the water to carbon mass energy absorption coefficient and mass collision stopping power ratios are constant, minimizing the need for considering corrections to energy fluence perturbations.^47^ Comparisons with ion chambers under photon, electron and proton beams for dose profiles, depth dose distribution, dose linearity, and dose rate independence, show that there is a similar or even better dosimetric response for synthetic diamond detectors.^65^, ^66^, ^67^

The low (near water) atomic number of all the structural elements of the diamond detector design is crucial for the reduction of any attenuation of the incident particles and for maintaining the energy independence of diamond. Based on previous studies,^68^, ^69^ there are variations of the diamond detector response that may be caused by irradiating high atomic number materials used for the conductive glue, metallic electrodes, encapsulation interface and wires. Diamond crystals have structural intrinsic symmetry, which means that the directional dependence of their sensitivity is minimal. However, encapsulation is once again critical, as it can compromise this characteristic, leading to an overall angular dependence on the response of the dosimeter.^40^

The performance of the only commercially established diamond dosimeter, mD, has been explored in numerous experimental and MC simulation studies for therapeutic beams.^17^, ^40^, ^64^ The device has a sandwich‐type metal/intrinsic diamond/p‐type diamond (m‐i‐p+) structure. An equivalent Schottky barrier junction is formed between the metal contact and the intrinsic diamond layer, resulting in a built‐in potential that enables the device to operate with zero applied bias.^61^ From these studies, its high spatial resolution and near tissue equivalence contribute greatly to the preferred application of this dosimeter in radiotherapy. The fact that this detector has a minimal directional dependence (with <1% deviation up to $\pm 60^{\circ }$) with high energy photons is also encouraging.^40^ From the above, as well as its other properties including energy independence, and negligible dose rate dependence with a deviation of less than ±0.5% up to 6 Gy/min,^40^ this commercial diamond dosimeter is categorized as a reference dosimeter in conventional radiotherapy dosimetry and has proven suitable, with certain limitations, for advanced radiotherapy techniques, as discussed in detail in the following sections.

Silicon diodes and diamond dosimeters have shown small differences in the determination of OFs, penumbras and field width evaluations and so, for routine beam commissioning and QA measurements where fields larger than 5 × 5 ${\mathrm{cm}}^{2}$ are required, there is no justifiable advantage in the use of diamond devices with respect to silicon diodes. One study^32^ supporting this statement is based on similarities observed in the beam profiles of the diode detector and the diamond detector in face‐on orientation to the beam. This is not the case though when delivering IMRT or SBRT treatments, where field sizes smaller than 2.6 × 2.6 ${\mathrm{cm}}^{2}$ are used. For such techniques, energy independence is a major requirement, heavily impacting the tail regions of the profiles, and silicon diodes lack this characteristic.^32^, ^37^ Consequently, diamonds are more suitable for use in stereotactic radiotherapy and small field dosimetry.

**FIGURE 1:**
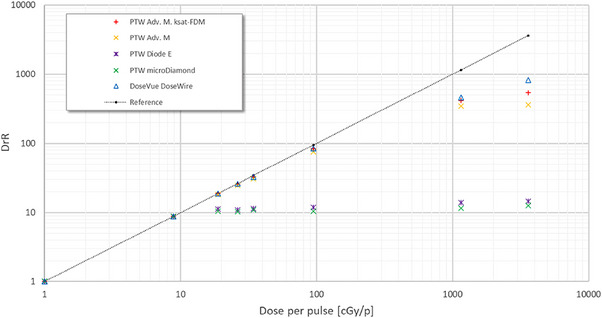
The dosimeter reading ratio, DrR, representing the ratio between the reading of each dosimeter when varying the DPP and its reading at 1 cGy/pulse, against DPP for different types of dosimeters. Radiochromic film measurements establish the linear fit.^18^

Solid‐state detectors appear to be more suitable for UHDPP approaches. In terms of the temporal resolution of these detectors (please refer to Table 2), diamond detectors have an improved timing response to silicon detectors, and there is less of a tendency for a charge collection efficiency dependence on the pulse repetition frequency (PRF) of the beam.^27^, ^58^, ^59^, ^70^, ^71^ However, for current commercially available solid state detectors used for clinical dosimetry, nonlinearity and DPP dependence exist in UHDPP.^18^, ^27^ Silicon diodes exhibit a nonlinear response with DPP (>0.15 Gy/pulse, τ = 2.5 μs), and, taking into account the effect of radiation damage in degrading the sensitivity, charge collection efficiency and leakage current,^72^ these detectors are described as being inadequate for FLASH radiotherapy.^54^ The results from a study conducted by Di Martino et al.^18^ support the findings described above and these are summarized in Figure 1 by comparing the DPP performance up to 40 Gy/pulse, with a τ value of 2.5 μs, for different types of dosimeters under electron beams. The dosimeters evaluated are an ionization chamber (PTW Advanced Markus electron chamber) with and without considering the correction factor for the ion recombination, ksat‐FDM, a silicon diode (PTW dosimetry diode E), a diamond dosimeter (PTW microDiamond), and a scintillator (DoseVue DoseWireTM Series 100 scintillating fiber). Measurements with radiochromic films (dose rate independent) are used to define the reference line. Despite the scintillator's higher cutoff value, ranging between 11 and 36 Gy/pulse, compared to the other investigated commercial dosimeters, all of them experience uncorrectable signal saturation in the FLASH region.^18^

Considering all of the above, the need for an accurate solid‐state active dosimeter that will overcome the challenges set by UHDPP and small field conditions is a major priority.

## 3 DIAMOND DOSIMETRY

### 3.1 Small photon beams

Dosimetry in non‐reference settings with non‐standard beams, particularly small fields, has grown in significance in this context. Specifically, the characterization of the mD for MV photon beams in small field dosimetry has been presented in several studies.^28^, ^29^, ^73^

The OFs with the mD compared to that with other detectors, such as radiochromic films, TLDs, silicon diodes and ion chambers, were evaluated in various studies for small radiotherapy beams with promising results.^37^, ^74^, ^75^, ^76^ For instance, with discrepancies less than 1% down to 1.6 × 1.6 ${\mathrm{cm}}^{2}$ and 1 × 1 ${\mathrm{cm}}^{2}$ field size, Zani et al.^76^ and Ciancaglioni et al.,^40^ respectively, produced OF values for FF beams that were in good agreement with those determined using small‐volume ionization chambers. Due to their smaller size, measured field penumbras with various diamond detectors as well as the mD are narrower than those obtained with the use of ion chambers.^31^, ^32^, ^66^ In fields smaller than 3 × 3 ${\mathrm{cm}}^{2}$, the mD's OF is very close to that of an unshielded stereotactic diode, and it is ∼3% lower than that of a shielded diode, whose overestimation has been discussed.^36^

The mD is also favorable over parallel and non‐parallel ion chambers when collecting depth dose measurements in the dose build‐up region for small field sizes, down to 0.6 × 0.6 ${\mathrm{cm}}^{2}$. It seems that different types of detectors have limitations when assessing the build‐up region but comparatively the most optimal detector for accurately representing the curve at all depths, with no surface correction down to 1 × 1 ${\mathrm{cm}}^{2}$ and a minimal correction of 0.6% at 0.6 × 0.6 ${\mathrm{cm}}^{2}$, is the mD.^77^ The detector choice in the build‐up region of small fields is extremely crucial for the data collected during beam commissioning, and hence accuracy of the dose distribution in a radiotherapy treatment plan. These findings are encouraging the use of the mD for pre‐treatment QA in special relevance to small field dosimetry.

Despite the above, there is an open debate in the literature on whether the mD detector is optimal for small field dosimetry, with inconsistent findings related to over‐response,^49^, ^73^ water equivalence,^78^ and under‐response for fields smaller than 2 × 2 ${\mathrm{cm}}^{2}$.^36^, ^48^, ^79^ The reason for the over‐response is that the elements surrounding the sensitive volume have a density higher than that of water causing the density perturbation correction factor to be less than unity and the detector to over‐respond.^17^ Based on multiple studies of the mD performance at the MV photon energy range,^28^, ^29^, ^73^, ^78^, ^80^ it is concluded that the high‐density perturbation effect remains dominant at small fields, investigated down to 0.5 × 0.5 ${\mathrm{cm}}^{2}$, leading to overestimations of OF values up to 7.8%.^28^ This is also evident at the crossline and inline profile regions outside the field, where correction factors reach a maximum of 11% down to 0.5 × 0.5 ${\mathrm{cm}}^{2}$, while density perturbations are reduced further down the tails.^48^ As a matter of fact, at large field sizes secondary electron fluence in the sensitive volume is not dependent on the density of the components surrounding it, due to secondary electron equilibrium.^69^ If the field size decreases and the beam half width is smaller than the range of lateral charged particle equilibrium plus half the size of the exterior volume of the detector, there will be less inward secondary electron movement across the field border, which will upset the secondary electron fluence. This will lead to a loss of charge particle equilibrium in central axis measurements.^1^

**FIGURE 2:**
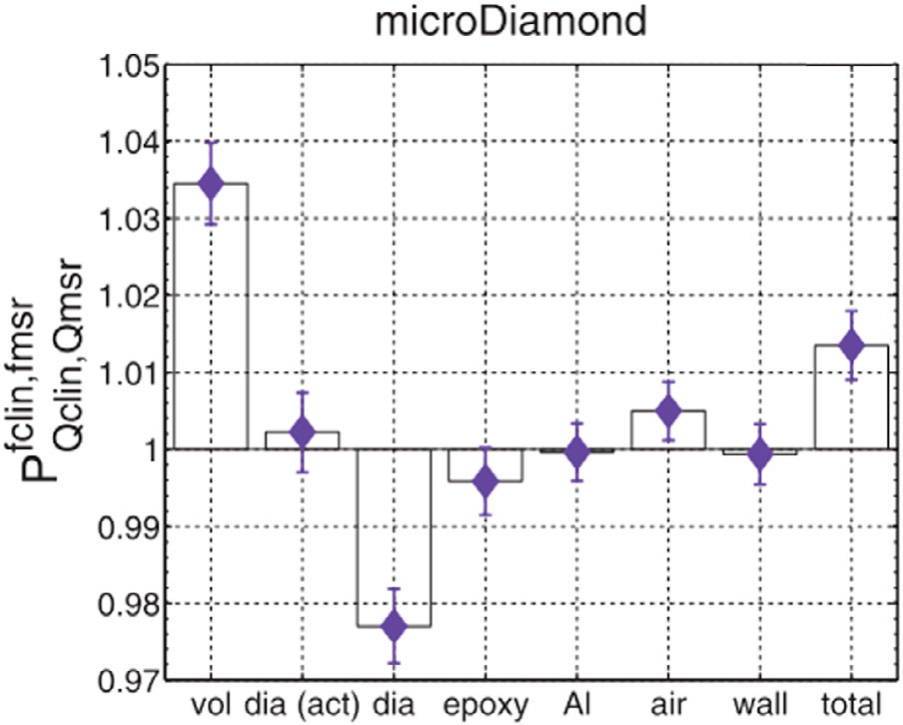
Individual contribution of each component of the mD detector to the total on‐axis relative perturbation factor, $P_{Q_{clin},Q_{msr}}^{f_{clin},f_{msr}}$, for a 0.5 × 0.5 ${\mathrm{cm}}^{2}$ field size. The active volume and the whole diamond component are represented by the dia(act) and dia legends, respectively.^48^

According to MC calculations and experimental work by Marsolat et al.,^33^ for diamond dimensions larger than approximately 1 × 1 × 0.3 ${\mathrm{mm}}^{3}$, the high density of diamond (3.52 times greater than that of water) results in an overestimation of the small beam OF. Although the thickness of the mD active volume is only 1 μm, for small beam OF measurements the volume averaging effect correction factors are still required due to its large lateral dimension of 2.2 mm, which is the threshold for maintaining a high signal‐to‐noise ratio (SNR). In a MC study, involving the mD, the OF and on‐axis region dose calculations for the profiles with a 0.5 × 0.5 ${\mathrm{cm}}^{2}$ field size were underestimated with an overall deviation of 1.3%.^48^ This was suggested to be the case due to the volume averaging effect and the air layers in the mD design dominating over other components, such as the epoxy and wall layers which individually lead to an overestimated response, as analytically presented in Figure 2. Palmans et al.^81^ support the idea that for fields <1 × 1 ${\mathrm{cm}}^{2}$ and for most detector types, volume averaging will have a larger effect in the $k_{Q_{clin},Q_{ref}}^{f_{clin},f_{ref}}$ values when compared to the effect produced by the material properties of the components of the detector, introducing a “turnaround” field size. The experimental and MC data presented in the literature do not indicate where this “turnaround” occurs with respect to field size, although it seems to be within 0.4 to 0.75 cm field widths^81^ and not exactly at 0.75 cm as stated in Das et al.^82^ Nonetheless, correction factors for volume averaging and density perturbation are needed for measurements of very small fields, <2 × 2 ${\mathrm{cm}}^{2}$.^16^, ^36^, ^48^, ^69^

The over‐response of the mD detector found in published MC research is frequently smaller than that observed in experimental tests.^73^, ^78^ Looe et al.^17^ suggest that the over‐response of this device for a small field size of 2 × 2 ${\mathrm{cm}}^{2}$ is due to the imbalance of radiation‐induced charge caused by the electrical contacts in the assembly. After considering the influence of charge imbalance in the experimental results of the mD, the difference between MC simulated and experimental $k_{Q_{clin},Q_{ref}}^{f_{clin},f_{ref}}$ values was reduced by shortening the metal contacts in the device. Based on these findings, a modified mD detector was built and investigated by Looe et al.,^17^ and it has demonstrated promising outcomes, with corrections smaller than 2% being required down to 0.6 × 0.6 ${\mathrm{cm}}^{2}$ fields. The disparity with experimental data is removed when this modification is applied to the MC data. MC and experimentally calculated correction factors may also differ due to a slight detector misalignment along the central axis. In a study by Alhakeem et al.^80^ detector alignment in the beam was within 0.1 mm and shifting the effective point of measurement off‐axis by this distance resulted in a signal loss similar to the observed inconsistencies between the calculated and measured OFs, up to 6.0% for 1.27 mm to 2.46 mm diameter cones and 3.7% for 3.77 mm diameter cones and above.

Additionally, the impact of the orientation of the detector has a crucial role in small field dosimetry. Face‐on (stem parallel) orientation to the beam is encouraged by TRS‐483 CoP^1^ for all small field QA measurements due to the potential asymmetries in the construction of the diamond dosimeters. It is important to remember that volume averaging has an impact on both orientations. In face‐on orientation, off‐axis components of the beam are averaged in the reading, and this becomes less critical as the field expands and the depth increases. In edge‐on (stem perpendicular) orientation to the beam, the measurement averages over a wider range of depths.^79^

Based on Brace et al.,^79^ in the build‐up region of PDD measurements for 1 × 1 ${\mathrm{cm}}^{2}$ and 3 × 3 ${\mathrm{cm}}^{2}$ fields, the response of a silicon diode, ion chamber and mD in face‐on orientation closely coincide. This suggests that the diamond and associated packaging do not cause any substantial disruption. In edge‐on orientation the volume averaging effect is negligible; the mD response is in agreement with that of the ion chamber at 5 and 10 mm depth for 1 × 1 ${\mathrm{cm}}^{2}$ and 3 × 3 ${\mathrm{cm}}^{2}$ fields, respectively.^79^ At 1 × 1 ${\mathrm{cm}}^{2}$ field size, the device in face‐on orientation has less of an over‐response than edge‐on. This is because face‐on orientation has a greater volume averaging effect leading to a lower OF.^79^ Overall, the overestimation for very small field sizes is evident in both face‐on and edge‐on orientations down to 1 × 1 ${\mathrm{cm}}^{2}$ field size.^79^ However, for much smaller field sizes a clearly improved performance is presented by Alhakeem et al.^80^ when it is placed edge‐on to the beam with the $k_{Q_{clin},Q_{ref}}^{f_{clin},f_{ref}}$ values reduced by 41.75% and 18.80% for a 0.127 and a 0.246 cm diameter cone, respectively.

Nevertheless, the angular dependence of both edge‐on and face‐on orientations should be assessed, especially when diamond detectors are employed for SBRT and VMAT.^75^, ^76^ For field sizes smaller than 2 × 2 ${\mathrm{cm}}^{2}$, the greater spatial resolution of the mD in edge‐on position is very beneficial. Down to this field size, the angular independence for $\pm 120^{\circ }$ is the primary benefit of placing the detector in an edge‐on orientation compared to face‐on orientation, with a maximum measured fluctuation of 2%.^79^ For advanced techniques, it is worth investigating the detector's orientation with respect to the beam, as angular dependence could require substantial correction factors. Therefore, the mD detector would be advantageous in edge‐on mode for QA of IMRT and VMAT.^75^

On a separate note, recent discrepancies between the results reported in the literature have led to some studies, such as the ones by Casar et al.^28^ and Das et al.,^82^ to question the recommended correction factors for the mD in TRS‐483 CoP.^1^ The published values for the mD detector $k_{Q_{clin},Q_{ref}}^{f_{clin},f_{ref}}$ values in TRS‐483 CoP^1^ are identical for 6 and 10 MV beams, which implies that there is no difference in $k_{Q_{clin},Q_{ref}}^{f_{clin},f_{ref}}$ regardless of the collimation system, filtration, beam energy and linac type. A study by Casar et al.^28^ suggests otherwise, for field widths <1 cm the mD detector's $k_{Q_{clin},Q_{ref}}^{f_{clin},f_{ref}}$ depends on the particular beam collimation system, beam energy and linac type utilized. This was established after measurements were performed with 6 MV FF, 6 MV FFF, and 10 MV FFF on an Elekta Versa HD linac. Statistically significant differences between $k_{Q_{clin},Q_{ref}}^{f_{clin},f_{ref}}$ values for all evaluated combination of beams at field widths of 0.5 and 0.8 cm were presented.^28^ Likewise, the study by Das et al.^82^ demonstrated that the difference between the $k_{Q_{clin},Q_{ref}}^{f_{clin},f_{ref}}$ values determined by various published studies for 6 MV, 0.5 × 0.5 ${\mathrm{cm}}^{2}$ fields delivered by either a Varian linac or the CyberKnife technology could reach up to 7% or 4%, respectively. On the contrary, another evaluation by Smith et al.^85^ demonstrated that reliable and consistent field output factor measurements can be achieved by following the protocol's recommended procedures. This was verified by comparing the field output factors of four mD detectors.^85^

**FIGURE 3:**
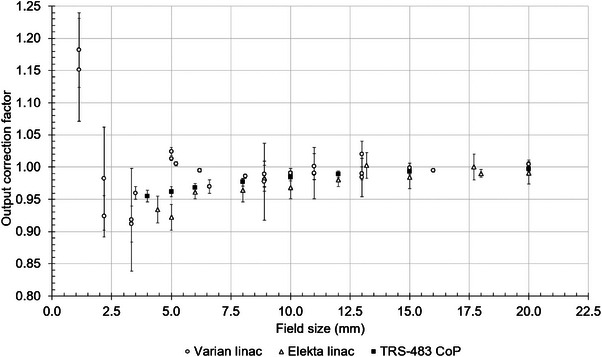
Summary of mD published $k_{Q_{clin},Q_{ref}}^{f_{clin},f_{ref}}$ values for Varian and Elekta linacs with 6 MV energy for small fields. The values reported in TRS‐483 CoP are represented separately as they are not distinguished between Varian and Elekta linacs.^1^, ^28^, ^29^, ^48^, ^49^, ^73^, ^80^, ^83^, ^84^

**FIGURE 4:**
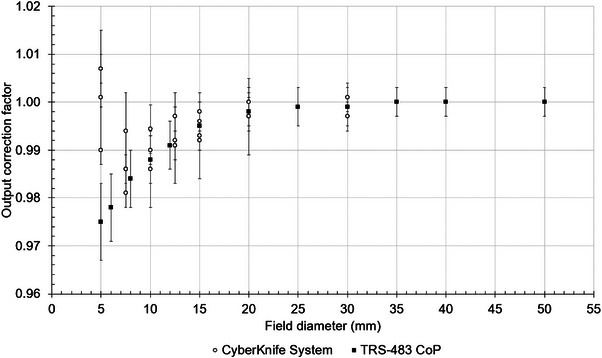
Summary of mD published $k_{Q_{clin},Q_{ref}}^{f_{clin},f_{ref}}$ values for a CyberKnife System with 6 MV energy.^1^, ^50^, ^74^, ^78^, ^83^

Figure 3 provides a compilation of the $k_{Q_{clin},Q_{ref}}^{f_{clin},f_{ref}}$ values from various published studies that includes MC simulations and experimental results from either Elekta or Varian linac with 6 MV energy. The data are derived from various depth and source‐to‐surface distance conditions and therefore the objective is to show a qualitative representation of the differences. Originally, some of the data are reported as a function of nominal field size, while other studies use the measured field size. Various $k_{Q_{clin},Q_{ref}}^{f_{clin},f_{ref}}$ values correspond to rectangular fields while others use circular collimators. For the purpose of Figure 3, the size of the field defined by the circular collimator was recalculated in terms of the equivalent square. Despite the above, some qualitative remarks can be made. It has been observed that for field widths of 10 mm and above, the values reported agree within 2.3%. When considering that the uncertainty of the values leading to this maximum difference is 1.7%, this difference is minimal. Moving to smaller field widths down to 5 mm, there are studies that indistinctively show an under‐response up to 2.4%^84^ and an over‐response up to 7.8% with respect to $k_{Q_{clin},Q_{ref}}^{f_{clin},f_{ref}}$ = 1.^28^ The largest deviation from the values reported in TRS‐483 CoP^1^ for the same range of field widths is 6.4%. For field widths below 4 mm, there is no data provided in the CoP. Overall, the largest $k_{Q_{clin},Q_{ref}}^{f_{clin},f_{ref}}$ value is observed at the smallest field width of 1.13 mm representing an underestimation of 18.2%.^80^ Similarly, in Figure 4 the $k_{Q_{clin},Q_{ref}}^{f_{clin},f_{ref}}$ values from various published studies using the CyberKnife machine at 6 MV energy are summarized down to 5 mm field diameter. The highest over‐ and under‐response observed, of 2.5%^1^ and 0.7%,^50^ respectively, occur at a field diameter of 5 mm. In general, the non‐homogeneity of the data below 10 mm is a result of the experimental set‐up, the reference detectors used to measure the OFs, MC simulation modelling and variations in the response of the mD.

Overall, a number of reasons for which diamond dosimeters are preferred in small field dosimetry have been presented. Similarly, some of the limitations and current debates about the response of the commercial diamond dosimeter in field sizes <2 × 2 ${\mathrm{cm}}^{2}$ are also discussed. Finally, an argument in favor of the use of the detector in the edge‐on orientation is outlined.^75^, ^79^, ^80^

### 3.2 FFF photon beams and FLASH radiotherapy

The combination of small fields and FFF beams is becoming more widely used in treatments such as SRS and SBRT as they continue to emerge, necessitating the use of sufficient tools for dose assessments.^86^ When exposed to FF and FFF photon beams the dose response of the mD in small fields, normalized to the response of alanine which is independent of dose rate and water‐equivalent in small fields,^22^, ^43^ agrees within the combined measurement uncertainties of less than 1%.^87^ This underlines the suitability of the diamond detector for small field dosimetry in FFF beams. The mD dose rate dependence with FFF beams is minimal even when exposed to high dose rate beams, up to ∼21 Gy/min, as characterized by Reggiori et al.^36^ Also, the effects of DPP dependence up to 2.2 mGy/pulse (τ = ∼4 μs) are proven to be negligible. Based on the same study, for both FFF and FF photon beams, the monitor unit (MU) rate dependence, dose linearity, and short‐term stability are <0.8%. When compared to results obtained with a reference ion chamber, OF values vary by <1% from 2 × 2 ${\mathrm{cm}}^{2}$ to 40 × 40 ${\mathrm{cm}}^{2}$ fields, and a commercial silicon diode, as expected, over‐responds by 3%.^36^ In addition, mD presents small differences, of less than 3.4%, in the dose response ratio between FF and FFF beams for field sizes down to 1 × 1 ${\mathrm{cm}}^{2}$.^86^ These findings, along with a very weak energy dependence, encourage the mD to be used for OF and profile measurements in FFF photon beams.

Furthermore, for FLASH, with UHDPP (>1 Gy/pulse) and microsecond pulses (typically ≥1 μs) with megagrays per second (MGy/s) instantaneous dose rates (I‐DRs), diamond detectors are very promising due to no ion recombination correction required and temperature independence. Most chemical vapor deposition (CVD) diamonds have a carrier lifetime of a few μs,^59^ hence with diamonds real‐time dose monitoring is indeed feasible. However, for natural and non‐commercialized CVD diamond detectors, a loss in sensitivity with dose rates is reported,^88^, ^89^ with clear effects on depth dose measurements. A first characterization of the mD in the UHDPP range was performed in a study by Di Martino et al.,^18^ where nonlinearity for DPP values greater than ∼0.15 Gy/pulse (τ = 2.5 μs) in electron beams is observed. This was also observed by Marinelli et al.,^90^ where this nonlinearity is shown to be related to the series resistance and sensitivity rather than being an inherent restriction of the detector's measurement mechanism. Measurements with two diamond prototypes confirmed that under high and UHDPP conditions using an ElectronFlash linac: (i) lowering the series resistance of the dosimeters, by increasing the boron concentration, and (ii) reducing the sensitive area to 1.5 ${\mathrm{mm}}^{2}$, results in a linear response up to at least 20 Gy/pulse (τ = 4 μs). Additionally, the PRF independence is maintained within ±0.5%.^90^ This firmly proves that a diamond dosimeter is feasible for FLASH radiotherapy applications.

**FIGURE 5:**
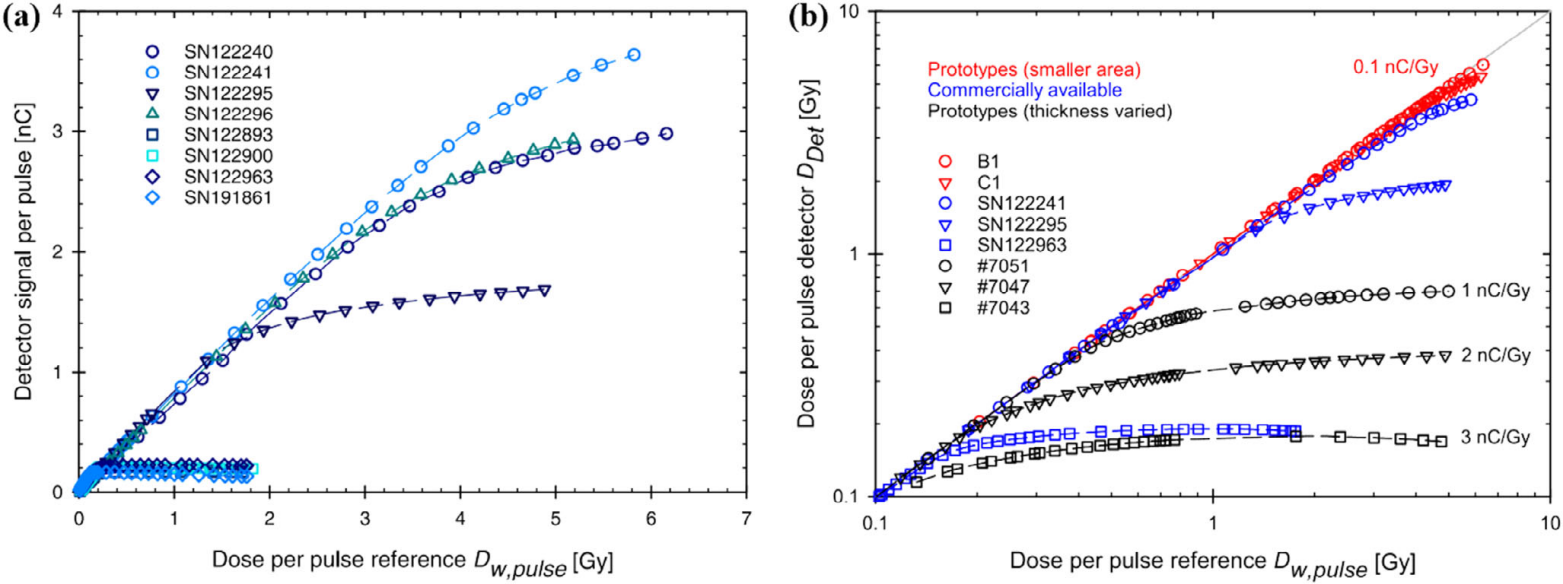
(a) Measured detector reading per pulse versus absorbed dose‐to‐water per pulse, $D_{w,pulse}$, at $z_{ref}$, for eight mD detectors, (b) Dose measured per pulse for three of the mD detectors presented on (a) and five prototypes created based on the mD structure with varied thickness and sensitive area dimensions, with respect to the reference $D_{w,pulse}$. The detector reading is converted to absorbed dose using the detector readings with and without radiation, the calibration coefficient for absorbed dose‐to‐water at 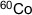 and the calibration correction factor with respect to the beam quality difference between measurements in 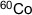 and the 20 MeV electron beam (τ = 2.5 μs).^91^, ^92^

A detailed investigation of the mD behavior in FLASH radiotherapy under a 20‐MeV electron beam was completed by Kranzer et al.^91^ Eight mD detectors were considered; the observed signal initially increases linearly with DPP and then a negative deviation from linearity becomes apparent for greater DPP values as shown in Figure 5a. The non‐linearity trend is followed by a saturation effect when DPP further increases and there is only a slight, almost non‐existent increase in the measured signal. The maximum charge per pulse, with a constant τ of 2.5 μs, detected determining the saturation level varies for each mD detector with a known sensitivity of ∼1 nC/Gy. For four of the mDs studied there was a maximum of 0.2 nC/pulse observed, three of the other mDs had a maximum of ∼3.8 nC/pulse and the other mD reached a maximum of ∼1.6 nC/pulse. It is safe to say that the linearity breaks off at DPP levels of ∼150 mGy/pulse for four of the mDs tested, ∼2 Gy/pulse for the other three and the last one falls between these two groups.

In order to determine the cause for such a difference in the mD's saturation behavior from device to device their diode characteristic curves were examined. The mD with the highest series resistance, suggesting a large voltage shift per small current change, had a lower saturation level than an mD with similar sensitivity. To clarify the impact of the series resistance, a further experiment was performed where an additional series resistance was added between the electrometer and the detector. As the resistance increased, the linear range and hence the maximum DPP acquired from the detector reading decreased. The series resistance for diamond detectors is mainly related to the metallic contact resistance of the top electrode, scattering and trapping effects in the intrinsic layer, the p‐type layer resistance, and the metal‐to‐p‐type interface contact resistance.^91^ To add to the above, the diode capacitance is also of relevance. During the pulse, an electric current is generated charging the diode capacitance and the signal cable connecting the detector with the electrometer. Diode capacitance discharge is delayed by high series resistance and this delay causes the diode to accumulate more voltage during the irradiation pulse. An increased DPP can raise this voltage to a threshold specified by the depletion region's intrinsic potential after which the detector responds nonlinearly to DPP because no charge carriers are separated beyond this value. Therefore, the curve describing the transition from the linear response to the saturation level depends on the series resistance, intrinsic potential, and diode capacitance.^91^

In the same study,^91^ eight prototypes were manufactured based on the mD structure, some with greater thickness dimensions, 2 and 3 μm, and hence sensitivities, and others with a much lower sensitivity, ∼0.1 nC/Gy, achieved by reducing the diameter of the sensitive area from 2.2 to 0.7 mm and keeping the thickness at 1 μm. The DPP performance of five of these prototypes and three of the eight mD detectors discussed earlier (one from each grouped response) is shown in Figure 5b. The prototypes with the larger sensitivity saturate at lower DPP values. For the two prototypes with the lowest sensitivity there is a linear response throughout the DPP range investigated, up to 6.5 Gy/pulse, with a deviation of up to 3%.^92^

Overall, the results from this study clearly show that saturation levels vary for the commercially available mD detectors despite their comparable sensitivity and that there is favorable behavior from lower sensitivity detectors. It is interesting to note that all detectors behave linearly up to 110 mGy/pulse, which corresponds to an I‐DR of 44 kGy/s, suggesting a promising potential for the mD to perform well under continuous proton beams with UHDR values. The results of this study are in agreement with the ones previously discussed by Marinelli et al.,^90^ suggesting that an optimal mD detector should have a low series resistance and a reduced sensitivity in order to eliminate non‐linearities under UHDPP electron beams.^91^

Following on from the studies discussed above, adjusting the active volume dimensions and boron concentration in the diamond doped layer transformed the commercial mD to a novel device referred to as the fD. This newly developed diamond‐based Schottky diode detector, with 1.4 mm active diameter and 1 μm thickness operating with no external bias voltage, was validated in pulsed electron beam dosimetry and used for commissioning the preclinical UHDR system, ElectronFlash.^93^, ^94^, ^95^ Measurements were performed at 7 and 9 MeV using PMMA applicators with a diameter ranging from 30 to 120 mm. The maximum I‐DR reached is 3 MGy/s, while maximum DPP is 13 Gy/pulse when τ is 4 μs.^93^, ^94^, ^95^

**FIGURE 6:**
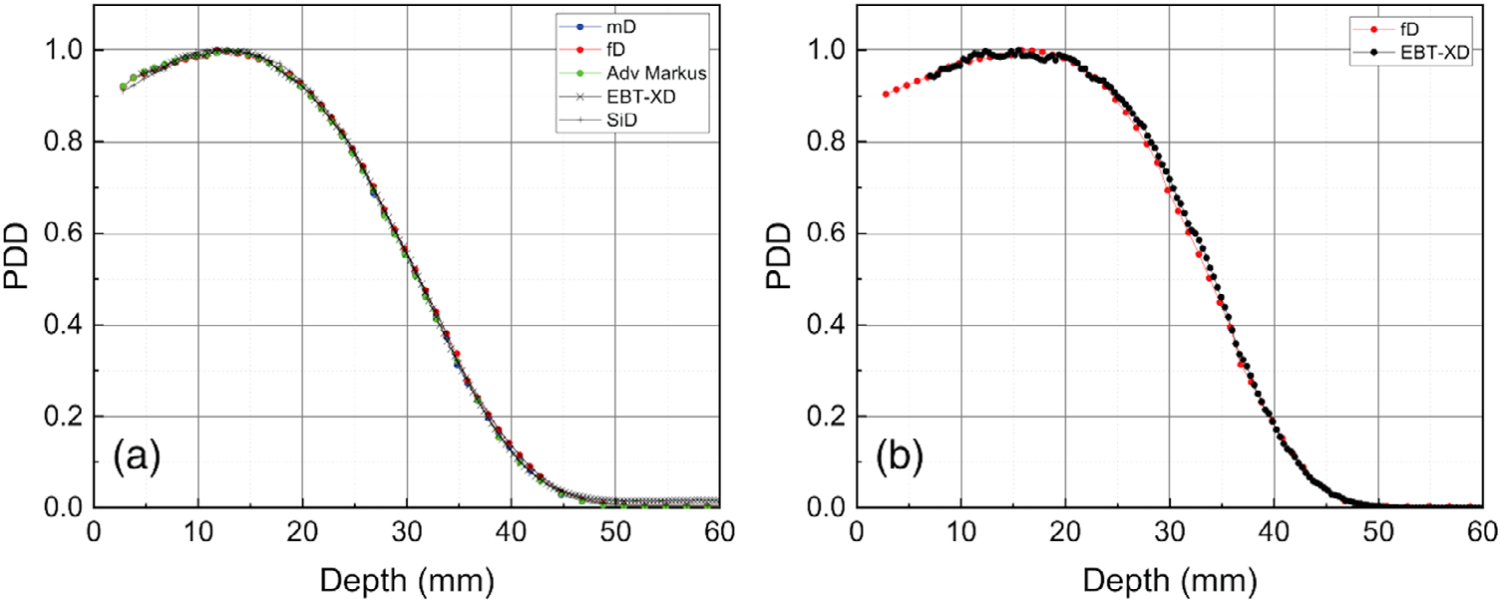
PDDs measured by the fD and the reference dosimeters under 9 MeV energy with a 100 mm PMMA applicator for (a) conventional and (b) UHDPP modalities.^95^

The fD was characterized in both conventional and UHDPP modalities and compared against various commercial dosimeters, including the mD under conventional beams and film dosimeters in UHDPP beams. A pre‐irradiation of 5 Gy is recommended to maintain a stable reading within 0.5%. The sensitivity of the fD, ∼0.3 nC/Gy, is independent of the beam energy and type when tested in 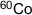, conventional electron beams and UHDPP electron beams. Excellent agreement, within the 5% uncertainty range, between the novel dosimeter and the reference dosimeters is presented for the beam profiles and OFs for both modalities. PDD measurements were acquired in less than a minute and correlate well with the reference dosimeters, with R90 and R50 variances of 0.5 mm in both beam modalities^95^ as shown in Figure 6. The results indicate that the fD can reliably measure dose linearly up to at least 13 Gy/pulse, within the uncertainty, while Gasparini et al.^93^ claim its potential to monitor levels up to 26 Gy/pulse, with τ equal to 4 μs. All in all, fD has been validated in conventional beams, and been characterized as suitable for UHDPP and UHDR measurements, presenting an innovative tool for commissioning UHDR devices in electron FLASH radiotherapy.^93^, ^94^, ^95^

The I‐DR generated during FLASH radiation treatments must be accurately measured to assess the “FLASH” effect in preclinical and biological studies. Marinelli et al.^96^ designed a system to accurately and comprehensively assess the temporal characteristics of electron UHDR beams with a time resolution of the order of a few tens of ns and experimentally determine the I‐DR and absolute dose values. A novel dosimetric system based on the fD device was created by replacing the standard PTW triaxial cable and plug with a coaxial version, and recording the signals by adding two instruments, a fast transimpedance amplifier and a digital oscilloscope as part of the electronics. In similarity with the constant sensitivity of the standard fD under different irradiation conditions discussed earlier,^95^ the calibration coefficient of this device in UHDR electron beams is estimated to vary by almost 1% with the measured value of 0.465±0.001 nC/Gy under reference conditions in a 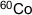 source. This is worth mentioning considering the difference in irradiation conditions, a 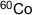 source with a 10 × 10 ${\mathrm{cm}}^{2}$ field and an ElectronFlash linac at 9 MeV electron beam energy with a 3 × 3 ${\mathrm{cm}}^{2}$ field. Also, integrating the I‐DR signals recorded by the modified fD yields linear absolute DPP values up to 7.13 Gy/pulse (τ = 4 μs), matching those of the standard fD. Marinelli et al.^96^ show that the modified fD is able to determine I‐DR up to 2 MGy/s with no saturation effects present, a good short term reproducibility of 0.9% with respect to pulse‐to‐pulse variation, and a slightly improved response time (no more than 90 ns) compared to the standard fD which is considered a negligible difference for microsecond pulses. Table 3 summarizes the irradiation conditions and DPP performance of the PTW mD and fD in electron beams from the studies discussed above.

**TABLE 3:** Summary of PTW mD and fD recent studies for electron UHDPP beams.

Study publication	Diamond dosimeter	Beam type	Max DPP	Max I‐DR	Pulse duration (μs)	DPP performance
Di Martino et al. (2020)^18^	mD	10 MeV electron beam	40.0 Gy/pulse	16.0 MGy/s	2.5	Non‐linear above 0.15 Gy/pulse
Marinelli et al. (2022)^90^	Prototypes based on mD structure	9 MeV electron beam	20.0 Gy/pulse	5.0 MGy/s	4	Linear within 5%
Kranzer et al. (2022)^91^	Prototypes based on mD structure	20 MeV electron beam	6.5 Gy/pulse	2.6 MGy/s	2.5	Linear within 3%
Gasparini et al. (2022)^93^	fD	7 and 9 MeV electron beam	13.0 Gy/pulse	3.0 MGy/s	4	Linear within 5%
Verona Rinati et al. (2022)^95^	fD	7 and 9 MeV electron beam	11.9 Gy/pulse	3.0 MGy/s	4	Linear within 5%
Marinelli et al. (2023)^96^	fD with altered signal transmission connection	9 MeV electron beam	7.1 Gy/pulse	2.0 MGy/s	4	Linear within 1%

Interestingly, in a study by Togno et al.^97^ the mD was tested under a UHDR millimeter‐small proton beam, as proposed in the study by Kranzer et al. mentioned above.^91^ This is the first study investigating the mD response in dose rates above 80 Gy/s in proton beams. In the past, mD has shown a dose rate response linearity within 3% up to 80 Gy/s in proton beams.^9^ For dose rates up to 2.2 kGy/s it is identified as capable of accurately measuring the dosimetric characteristics of narrow pencil proton beams in real time within just 2% dose rate dependence.^97^ Further investigation regarding a certain dose rate saturation point in proton beams, similar to that encountered in UHDR pulsed electron beams,^91^ should be conducted.

Although this review does not focus on the performance of dosimeters in the medium‐energy x‐ray range (as not considered advanced radiotherapy modality), it is noteworthy that the mD detector has been characterized in MRT beams with a mean energy of 95 keV and up to 700 Gy/s dose rate. Due to the high spatial resolution of the detector in the perpendicular orientation, the microbeams are well‐resolved in the profile measurements and the PDDs obtained agree with those measured by an ionization chamber within 2%.^98^ Moreover, the efficacy of a microstriped diamond portal detector for real‐time readings in MRT beams has been demonstrated up to 1.2 × 10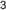 Gy/ s.^99^

In conclusion, the mD offers precise measurements of OFs for the whole spectrum of field sizes commonly used in radiotherapy, >2 × 2 ${\mathrm{cm}}^{2}$, without the need for correction factors in FFF photon beams. Yet, this dosimeter and other commercially available semiconductor dosimeters are not optimal for electron FLASH conditions and are still under investigation for proton FLASH beams. The proposal of the fD as a modified version of mD has been presented as optimal for dosimetric measurements in UHDPP and valuable for commissioning UHDR electron beam systems, however MC simulations are still in progress for the theoretical validation of the device.^18^, ^95^, ^97^

### 3.3 Proton and hadron therapy

In proton and hadron beam therapy, a major challenge is finding high spatial resolution dosimeters with sufficient energy and dose rate independence. A pre‐commercial prototype and the commercial version of the PTW mD were tested with therapeutic proton beams.^64^, ^100^, ^101^, ^102^ According to these studies, when proton beams are incident to the device a minimal dose rate dependence tested up to 3 Gy/s and energy dependence from 70 to 230 MeV is exhibited. Specifically, in the work of Akino et al.^100^ the experimental findings show that the depth dose curves measured with the diamond device and a plane‐parallel ionization chamber are in good agreement. A linear response to absorbed dose is observed for the mD down to ∼0.03 Gy and up to 5 Gy with deviations below 0.5%.^64^, ^102^ Interestingly, the study by Marsolat et al.^101^ demonstrates a lack of reproducibility for two of the four mDs characterized in this work in terms of sensitivity, stability, and LET dependence. In a clinical 138 MeV passive scattering proton beam, there is a maximum difference in the peak‐to‐plateau ratio of 6.7% determined by the mDs and the reference ionization chamber, and a variation of ∼10% for the Bragg peak dose across the four detectors.^101^ The reported discrepancies on the response of mD detectors in the Bragg beak implies a lack of reproducibility to assess the absorbed dose in proton beam dosimetry.

LET dependence is the main limitation of high spatial resolution detectors in accurately measuring the Bragg peak in proton beams, alongside other particle therapy techniques. Studies on the mD detector present inconsistent findings regarding the LET dependence of the device. The LET independence for a synthetic diamond detector, based on the mD assembly before being commercialized, is highlighted in a study by Mandapaka et al.^64^ evaluating depth dose curves for high‐energy proton beams, ranging from 126 to 250 MeV. Comparing the depth dose curves of the detector to the ones of a reference ionization chamber, the difference in the peak‐to‐plateau ratios is at most 2.3%.^64^ It is worth mentioning that natural diamond detectors used in the past showed a considerable depth dependence underestimating the Bragg peak as mentioned in the studies by Fidanzio et al.^103^ and Sakama et al.^104^ The variation in the response of natural diamond detectors and the mD, a synthetic diamond detector, seems to be related to the smaller sensitive volume thickness of the mD reducing the space charge formation and carrier recombination effects.^64^ Based on Goma et al.^102^ and Rossomme et al.^105^ the mD also shows negligible LET dependence under lower proton energy beams, of 70 and 62 MeV, respectively. In both studies the point detector is compared to a reference ionization chamber and a good agreement is demonstrated between the two detectors at the distal edge and the plateau regions.

One study contradicting the negligible LET dependence of the mD in proton beams is that by Marsolat et al.,^101^ which was discussed previously. The depth dose curves for two proton beam energies, 89 MeV and 138 MeV, were investigated and compared to the curves measured with a reference ionization chamber. For both beam energies one of the mDs presents an underestimated dose, up to ∼2.8%, in the Bragg peak region, while another overestimates the dose in that region, up to ∼6.7%. The other two devices have a maximum peak‐to‐plateau ratio difference to the reference ionization chamber of 2.3% and 1.8%, respectively, for the two beam energies. The latter two mDs have a minimal LET dependence in contrast to the first two devices. Apart from that, another interesting investigation conducted in the same study is the orientation of the detector to the beam source. As indicated, the edge‐on orientation of the detector to the beam direction leads to an underestimated dose value of 8.1% for the Bragg peak and a wider Bragg peak width. This is suggested to be related to the volume averaging effect caused by the larger sensitive volume dimension (2.2 mm compared to 1 μm when face‐on to the beam direction) in this orientation.^101^

Less debatable is the response of the mD at higher LET values achieved with carbon or oxygen ion beams. When exposed to a 62 MeV/n monoenergetic carbon ion beam, the mD showed an LET dependence.^106^ Comparing the mD response to that of an ionization chamber, as a ratio, in the plateau region of the Bragg peak curve, a strong correlation was found, with the ratio decreasing in the Bragg peak region and the mD under‐responding by ∼20% at the distal edge region. The relative standard uncertainty was 2.3% in the plateau and 12% in the distal edge.^106^ To ensure this was not a single detector's biased response, another study found a similar under‐response of ∼13% for multiple mDs in carbon and oxygen ion beams of the same energy.^107^ On the contrary, Marinelli et al.^108^ found the detector's response independent of LET in a 280 MeV/n carbon ion beam. This finding does not necessarily conflict with the data presented in a mono‐energetic, non‐modulated, low‐energy beam by Rossomme et al.^106^, ^107^ This difference might be due to the broader LET spectrum in the Bragg peak region for higher energy beams, with further range modulation employing a ripple filter, which could hide sensitivity reductions to high LET values.

**FIGURE 7:**
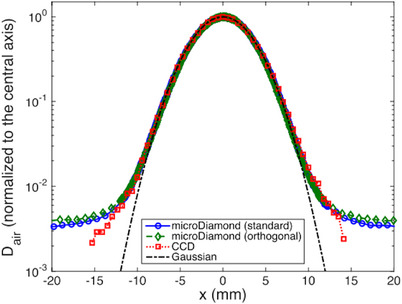
Standard and orthogonal orientated mD lateral dose profiles when placed at the isocenter, in air, for a 150 MeV proton pencil beam. CCD camera data are not to be referred to for any conclusions and the Gaussian distribution is included for illustrative purposes.^102^

In terms of profile measurements performed with the mD in proton beams, there is also a detector orientation dependence. Goma et al.^102^ compared the profile dose curves of the mD placed in orthogonal (edge‐on) versus standard (face‐on) orientation to a 150 MeV proton pencil beam as illustrated in Figure 7. In the orthogonal orientation, there is an over‐response at the tail regions of the profile due to the greater build‐up region of the detector (∼7 mm) compared to the standard one (∼1.1 mm). Low‐energy protons are strongly affected by this due to the steeper dose gradient at the entrance window compared to high‐energy protons. The distribution presented in the tail regions is created by low‐energy protons, scattered by large angles from the nozzle, which in this case end up being overestimated.^102^

Following on from the mD limitations, the SCDD‐Pro prototype by Moignier et al.^109^ was built with a front silver resin and a diamond crystal that is 150 μm thick and 1 mm wide. To assess how well this diamond detector operates in a medical setting, it was tested with broad, passive scattering proton beams and pencil beam scanning proton beams with energies between 100 and 220 MeV. More generally, it is shown that it is the density of the diamond crystal that mainly affects the depth‐dose curves and not the atomic composition. Depending on the beam energy and diamond thickness, the Bragg peak dose for the crystal alone is routinely underestimated.^109^ According to MC simulations, this perturbation can be corrected by placing a certain element, in this case a silver resin, in front of the active volume. Experimental evidence demonstrates that, for all of the tested proton beams, the deviations in the depth‐dose curves and the Bragg peak dose are both below 1.6% and 0.3%, respectively. In addition, the repeatability, dose rate dependence between 1 and 5.5 Gy/min, and linearity for doses >0.05 Gy are better than 0.4%.^109^ From these results, it is possible to conclude that this device lowers the error on the Bragg peak dose and is appropriate for precise depth‐dose measurements for proton pencil beams of 5 mm in full width half maximum and high energies.^109^ The use of another CVD diamond assembly for proton beam dosimetry is investigated by Cirrone et al.^67^ where the detector's response for dose and dose rate is characterized as stable for 62 MeV proton beams, reinforcing the suitability of CVD diamonds for proton beam dosimetry.

More recently, in a study by Kretschmer et al.^110^ discrepancies are presented in the mD profile distribution with a 150 MeV scattered proton beam collimated by a 0.5 mm wide slit collimator. There is a signal drop of 1.9% and 3.9% observed in photons and protons respectively. Due to the shorter average secondary electron range in proton beams,^111^ and as a result of density perturbations, the over‐response along the beam axis and the steepening of the profile at the beam penumbra regions are expected to be less pronounced. This finding is consistent with the profiles simulated in the study discussed earlier by Moignier et al.,^109^ in which the field size and lateral penumbra width deviations are larger than 0.17 and 0.08 mm, respectively, when increasing the width of the SCDD‐Pro prototype from 1 to 2 mm or greater.^109^

Summarizing the key points presented above, the application of diamond detectors in proton and hadron therapy seems promising with emphasis on the energy and dose rate independence. However, the commercially and clinically established diamond dosimeter is non‐optimal for measurements in this type of beams, emphasizing the need for a new diamond detector device. In addition, as shown by various studies above, the dimensions and composition of the diamond detector design have a large impact on its dosimetric behavior.

### 3.4 Other developments

The challenges presented by the continuous introduction of modern radiotherapy techniques have driven researchers to explore alternative structural forms of diamond detectors that can be advantageous. The “3D” configuration of a diamond dosimeter introduces a new pathway to overcome the limitations of planar diamond detectors in small field dosimetry. Besides this, the temporal resolution of diamond detectors can be extremely beneficial for dose‐per‐pulse beam front‐end readout systems.

#### 3D diamond detectors

**FIGURE 8:**
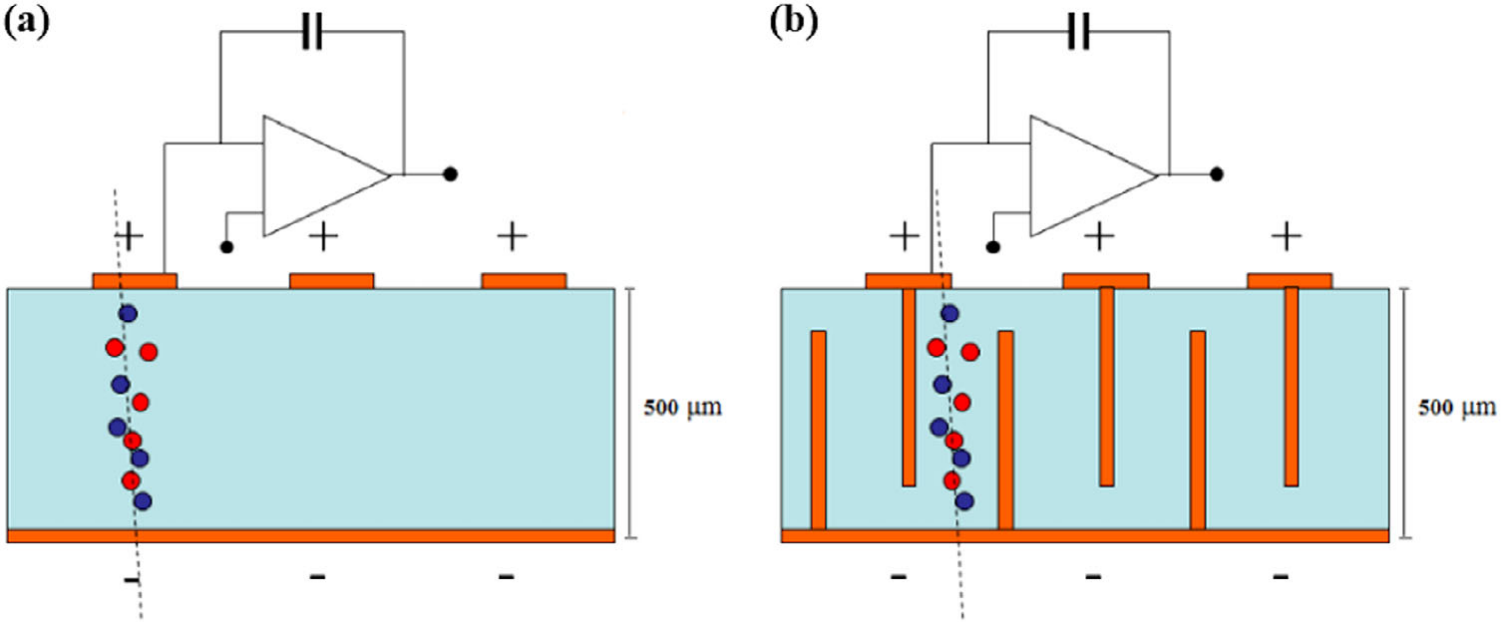
Operative structure of (a) a planar diamond detector and (b) a 3D diamond detector. The diamond volume is represented in blue and the graphite electrodes in orange.^112^

Apart from planar diamond devices, there is much recent interest in 3D diamond devices, produced by a pulsed laser technique which creates graphitic pathways inside a polycrystalline (pCVD) diamond. Thin, cylindrical electrodes are placed perpendicularly to the sensor surface a few tens of microns apart, which is less than the charge collection distance, restricting the generated charge to a single cell with electrodes at its vertices. A schematic of this structure and the structure of a planar diamond detector is shown in Figure 8. Due to the reduced polarizing electrode spacing, the “3D” geometry requires a lower bias voltage to achieve the saturation charge velocity, consequently leading to a greater charge collection efficiency, faster detector response and greater suitability for radiation‐intensive environments.^72^, ^112^, ^113^ Using a 500‐μm thick diamond, a 3D diamond detector can accumulate charge particles with a bias voltage 10 times less than that required for a 2D sensor.^72^

In addition, the high spatial granularity of such a device allows the spatial dose distribution to be evaluated more accurately than for planar devices. For instance, to establish a comprehensive dose profile, additional measurements need to be taken to improve the positional accuracy for mD, but each measurement's uncertainty contributes to an increase in the overall uncertainty of the final measurement. To address this, it is anticipated that 3D diamond dosimeters will incorporate a larger surface area with dimensions of 2.5 × 2.5 ${\mathrm{cm}}^{2}$, feasible with pCVD diamonds, enhancing segmentation capabilities.^112^

Very promising performance of 3D diamond devices is observed in high‐energy photon beams.^112^, ^113^ A 3D device with a single cell sensitive volume of 0.004 ${\mathrm{mm}}^{3}$ and a total sensitive volume of 1.1 ${\mathrm{mm}}^{3}$, at an operating bias voltage of ‐80 V, showed a linear dose response within 2%, a stability as good as 0.6%, and an energy dependence below 3% for energy beams in the range of 6 to 10 MV. Additionally, it has the capability of assessing the beam flatness and full width half maximum for a 10 × 10 ${\mathrm{cm}}^{2}$ field size with a high level of precision of 1% compared to measurements with GafChromic films. Even though it consists of a pCVD diamond, there is a high sensitivity of 121.3±0.2 nC/Gy observed when placed in a PMMA phantom with an energy of 6 MV and 5 × 5 ${\mathrm{cm}}^{2}$ field size. These studies are encouraging for the application of 3D diamond devices in small fields, considering as well that the beam direction dependence and profile correction factors would be minimized. The latter occurs by conducting simultaneous measurements at various locations within the beam shape and penumbra region.^112^, ^113^

Building upon the aforementioned, Talamonti et al.^114^ investigated the suitability of 3D diamond devices in small field dosimetry. Relative dose measurements for the signal ratio in field sizes of 1.6 × 1.6 ${\mathrm{cm}}^{2}$ to 10 × 10 ${\mathrm{cm}}^{2}$ were taken for a 3D diamond device in a PMMA phantom at 6 MV energy. These measurements are compared to the point detector mD and there is a remarkable agreement of 0.5% down to the smallest field size. In addition, it responds linearly to dose within 2%, and the time stability and repeatability are excellent under these conditions. Such findings could form the basis for the development of a new diamond‐based QA dosimeter device.^114^

More recently in the work by Kanxheri et al.,^115^ the impact of the 3D pixel size on various dosimetric parameters, including absorbed dose sensitivity, linearity with dose, reproducibility, and beam profile measurements, was evaluated in high‐energy photon beams with small field sizes. All measurements were performed in a 1.6 × 1.6 ${\mathrm{cm}}^{2}$ field, except for the beam profile distribution which was obtained in a 0.8 × 0.8 ${\mathrm{cm}}^{2}$ field. Multiple pCVD diamond detectors were manufactured with a 2.5 × 2.5 ${\mathrm{cm}}^{2}$ surface area and 500 pixels with graphitic electrodes, and metal pads on the detector's surface used for wire bonding between the 3D pixels and the PCB. The detectors tested had sensitive volumes ranging from 0.080 to 0.281 ${\mathrm{mm}}^{3}$, all with the same thickness of 500 μm and varying active areas, operated at +10 V. All of the devices responded independently to dose rate, within 1%, up to the maximum operated value of 4.7 Gy/min. Pixel sensitivity decreased with size, yet even the smallest pixel (0.16 ${\mathrm{mm}}^{2}$ active area) had a high absorbed dose sensitivity of 15 nC/Gy. Nevertheless, pixel size is critical for beam profile measurements. The smallest pixel size led to the most consistent agreement of the penumbra width between the tested detector and the mD. The air gap above the detector, the backscattering from metallic electrodes and the volume averaging effect of larger sized pixels perturb the beam profile causing an over‐response outside the field edge.^115^

The latest 3D diamond device studied and published^116^ aims to reduce any perturbations raised in dosimetric measurements by the metal‐diamond interactions as mentioned in the past by Kanxheri et al.^115^ This is the first 3D all‐carbon diamond detector with laser‐inscripted graphitic surface connectors and bonding pads.^116^ Based on a pCVD with a smaller surface area of 0.5 × 0.5 ${\mathrm{cm}}^{2}$ and the same thickness as the previous devices, an active pixel volume of 0.14 ${\mathrm{mm}}^{3}$ is created. At a preliminary stage, testing this device with 6 MV energy resulted in good performance, including a dose rate independence, similar to other 3D diamond devices^16^, ^112^, ^113^, ^115^ when compared to the mD. The removal of metal interfaces close to the diamond surface allows it to operate at a lower bias voltage, down to +6 V.^116^ The development of all‐carbon 3D diamond devices is to be explored further in the following years.

#### Diamond‐based dose‐per‐pulse x‐ray detectors

Rapid X‐ray detectors and reliable beam front‐end readout electronics help treatment plans meet QA requirements. Ionization chambers are commonly used to measure the dose delivered having usually a collection time of a few ms, generally ranging from 10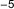 to 10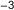 s.^24^, ^57^ They are connected to electrometers that have notably longer integration durations, typically between 0.1 and 10 s,^117^ and consequently the acquisition of pulse‐by‐pulse measurements at high repetition rates is not feasible. Recently, a detection system was created that employs a high‐quality single crystal diamond dosimeter with MSM (metal‐semiconductor‐metal) structure, connected to a customized front‐end readout electronic system that amplifies/attenuates the signal.^41^ This system aims to quantify and analyze the dose of each pulse emitted by a linac with 6 MV energy beams, tested up to 10 Gy. The charge for a single pulse recorded was 84.68 pC, a value which is consistent with the estimated value from a commercial electrometer in continuous integration mode. This device performs real‐time dose‐per‐pulse monitoring, with a rapid response time of a few ns, reducing the inaccuracies caused by large acquisition times.^41^

In the past year, a similar diamond‐based dose‐per‐pulse x‐ray detector has been characterized under various beam energies and sources to assess its suitability under different radiation environments.^118^ An excellent linear response with dose, and dose rate independence under low‐energy continuous x‐rays (up to 40 keV), 6 MeV energy pulsed x‐rays as well as 6 MeV pulsed electron beams, was observed. The sensitivity of the device was not perturbed with respect to the radiation type, with a sensitivity of 0.299 ± 0.002 μC/Gy in 6 MeV x‐rays and 0.298 ± 0.004 μC/Gy for 6 MeV electrons. The sensitivity under 6 MeV X‐rays is 0.15 times the sensitivity at low‐energy x‐rays (1.988 ± 0.005 μC/Gy), which is equivalent to the ratio of the mass attenuation coefficients of the two x‐ray energy beams in carbon material, published by the National Institute of Standards and Technology (NIST). Hence, this study showcases the notable advancements achieved in CVD diamond technology, which enable the production of highly versatile detectors appropriate for both intra‐ and extra‐operative radiotherapy methods, along with their application in radiation protection dosimetry.^118^

The progress in the diamond dosimetry industry is enabling the application of diamond substrate‐based detectors in advanced radiotherapy techniques for precise dosimetry measurements.

## 4 CONCLUSIONS

Small fields are at the forefront of new radiotherapy treatment modalities, like IMRT, VMAT, SRS, and SBRT. At the same time, new modalities with very high and UHDRs are being introduced. For each of these, the development of new reliable detectors will improve the confidence in the implementation and delivery of such techniques. In this review, all types of dosimeters discussed seem suited to the requirements of small fields, each one of them with its own advantages and disadvantages which need to be carefully considered. Single‐crystal diamond dosimeters could be the detector of choice for measurements of reference and relative absorbed dose in beams delivered by these techniques, due to their high spatial and temporal resolution, negligible ion recombination effect, near tissue equivalence, and minimal energy dependence. However, the components surrounding the sensitive volume of diamond, such as the housing and PCB choice, the read‐out system, and the size of the sensitive volume, all crucially perturb the dose measured. The commercially established mD has limitations in small fields <2 × 2 ${\mathrm{cm}}^{2}$, UHDPP, proton and hadron therapy. For field sizes <1 × 1 ${\mathrm{cm}}^{2}$ there is high variability in the $k_{Q_{clin},Q_{ref}}^{f_{clin},f_{ref}}$ values reported by MC and experimental published studies, presenting a competition between the dominance of volume averaging and density perturbation effects. PTW has recently introduced the fD as an altered version of the mD that is promising for DPP values up to at least 20 Gy/pulse (τ = 4 μs), but MC simulations supporting the validation of this device are still to be completed. Finally, researchers have investigated the applicability of diamond detectors in other forms, such as through pCVD 3D configurations providing great advantages in small field dosimetry and as real‐time dose per pulse x‐ray detectors with high temporal resolution. All in all, recent developments in novel diamond dosimetric devices explore new pathways attempting to address the challenges encountered in the dosimetry of advanced radiotherapy techniques.

## CONFLICT OF INTEREST STATEMENT

The authors have no relevant conflicts of interest to disclose.
